# The Cardiovascular Therapeutic Potential of Propolis—A Comprehensive Review

**DOI:** 10.3390/biology10010027

**Published:** 2021-01-04

**Authors:** Henrique Silva, Rafaela Francisco, Ariana Saraiva, Simone Francisco, Conrado Carrascosa, António Raposo

**Affiliations:** 1 Informetrics Research Group, Ton Duc Thang University, Ho Chi Minh City 758307, Vietnam; 2 Faculty of Pharmacy, Ton Duc Thang University, Ho Chi Minh City 758307, Vietnam; 3 Pharmacological Sciences Department, Faculty of Pharmacy, Universidade de Lisboa, Av Prof Gama Pinto, 1649-003 Lisboa, Portugal; rafaelafrancisco@campus.ul.pt; 4 Department of Animal Pathology and Production, Bromatology and Food Technology, Faculty of Veterinary, Universidad de Las Palmas de Gran Canaria, Trasmontaña s/n, 35413 Arucas, Spain; ariana_23@outlook.pt (A.S.); conrado.carrascosa@ulpgc.es (C.C.); 5 Faculty of Medicine, Nutrition Lab—Universidade de Lisboa, 1649-028 Lisboa, Portugal; on1948@onutricionistas.pt; 6 CBIOS (Research Center for Biosciences and Health Technologies), Universidade Lusófona de Humanidades e Tecnologias, Campo Grande 376, 1749-024 Lisboa, Portugal

**Keywords:** propolis, antihypertensive, antiangiogenic, antioxidant, cardiovascular protection

## Abstract

**Simple Summary:**

Propolis, also described as bee glue, is a natural component made up of a resinous mixture of honeybee compounds from multiple botanical sources. The literature has demonstrated a variety of medicinal properties attributed to propolis due to its chemical complexity. However, the positive effects of propolis on cardiovascular health have gained little coverage. Therefore, we aimed to provide an accurate and up-to-date review of the main cardiovascular health benefits of propolis. In particular, we intend to establish the key varieties of propolis and pharmacological compounds with the therapeutic effects that are most encouraging, as well as the physiological processes by which those advantages are accomplished. The Brazilian green and red varieties reveal the greatest number of beneficial activities among the varieties of propolis studied. While much of the cardiovascular beneficial effects appear to derive from the cumulative actions of several compounds working via multiple signaling mechanisms, some individual compounds that may enhance the existing therapeutic arsenal have also shown significant results. It is also worth exploring the prospect of using propolis as food supplements.

**Abstract:**

Owing to its chemical richness, propolis has a myriad of therapeutic properties. To the authors’ knowledge, this is the first comprehensive review paper on propolis to focus exclusively on its major effects for cardiovascular health. The propolis compound varieties with the most promising therapeutic benefits and their respective physiological mechanisms will be discussed. Propolis displays an anti-atherosclerotic activity, attained through modulation of the plasma lipid profile and through stabilization of the fatty plaque by inhibiting macrophage apoptosis, vascular smooth muscle proliferation and metalloproteinase activity. The antihypertensive effects of propolis probably arise through the combination of several mechanisms, including the suppression of catecholamine synthesis, stimulation of endothelium-dependent vasorelaxation and vascular anti-inflammatory activity. The anti-hemostatic activity of propolis is attributed to the inhibition of platelet plug formation and antifibrinolytic activity. By inhibiting the secretion of proangiogenic factors, propolis suppresses endothelial cell migration and tubulogenesis, exerting antiangiogenic activity. The antioxidant and anti-inflammatory activities are responsible for protection against vascular endothelial and cardiomyocyte dysfunction, mostly by the prevention of oxidative stress. Among the reviewed propolis varieties, the Brazilian green and red varieties show the largest number of beneficial activities. Further research, especially preclinical, should be conducted to assess the cardiovascular benefits of the given varieties with different compositions.

## 1. Introduction

This paper intends to provide an objective and up-to-date comprehensive review on propolis. To the authors’ knowledge, this is the first paper to focus exclusively on its cardiovascular effects, covering more subjects than the most recently published reviews [1,2]. Propolis, also known as bee glue, is a natural product consisting of a resinous mixture of substances collected by honeybees (*Apis mellifera* L.) from different botanical sources, beeswax and salivary enzymes. “Propolis” is a Greek word meaning “defense of the city”, which alludes to the fact that honeybees use propolis as a sealant material. At the beehive entrance, propolis traps predators, being used also to fill in the beehive cracks and as a thermal isolator, therefore maintaining the hive in perfect health [3,4]. From a physicochemical standpoint, propolis is a lipophilic material, hard and breakable when cold but soft and very sticky when warm. It has a pleasant aromatic smell and a variable coloration, which depends on its botanical source, age and geographical climate, ranging from cream, green or red to brown [4,5]. Table 1 summarizes the main propolis geographic origins and their major constituents.

Propolis has been used as a folk medicine since 300 BC owing to the multitude of its beneficial health effects [47,48], which have motivated a vast array of applications in several health sciences. It has been used as an ingredient in dental hygiene (e.g., toothpastes and mouthwashes) and cosmetic (e.g., creams and lotions) products, as well as a chemical food preservative [49,50]. The first scientific report on propolis was published in 1908, detailing its composition and chemical actions [51,52]. Propolis contains many dozens of compounds, mainly polyphenols, resins, flavonoids, essential oils, fatty acids, wax, pollen and other organic substances [53,54]. It also comprises vitamins (B complex, C and E) and relevant minerals such as zinc, magnesium, copper, iron, manganese, nickel and calcium [55,56]. Owing to its chemical richness, propolis has a myriad of therapeutic properties, although the best studied are antioxidant, anti-inflammatory, immunomodulatory, antibacterial, antiviral, antifungal and antiproliferative [4,57]. In contrast, little attention has been given to the beneficial effects of propolis in cardiovascular health. In this paper, the authors identify the main propolis varieties and pharmacological compounds with the most promising therapeutic benefits, as well as the physiological mechanisms through which those benefits are attained.

## 2. Cardiovascular Effects of Propolis

Cardiovascular diseases are the number one cause of mortality globally, representing a high economic burden and deeply impacting the patients’ quality of life [58,59,60]. Given the continuous need for new drugs and especially considering the dependence of developing countries on traditional medicines [61], a clarification on the cardiovascular benefits of propolis is warranted. As reviewed in this paper, the cardiovascular effects of propolis are attributed to a combination of several biological activities such as antioxidant, anti-inflammatory, immunomodulatory, antihypertensive, anti-atherosclerotic and antiangiogenic, which are often interrelated with each other. The next sections detail the main cardiovascular effects of different varieties of propolis.

### 2.1. Anti-Atherosclerotic Effect

Atherosclerosis is a chronic condition characterized by the progressive hardening and narrowing of arteries, consequently reducing perfusion to organs. It is caused by the accumulation of fatty plaques in their inner wall, consisting of low-density lipoprotein (LDL), cellular waste and surrounding materials [62]. High LDL and low high-density lipoprotein (HDL) levels are important risk factors. In atherosclerosis, the vascular endothelium becomes permeable to the passage of serum lipids, especially LDL, into the arterial wall. In the subendothelial space, the oxidation of LDL activates endothelial cells into producing adhesion molecules, leading to the recruitment of circulating monocytes and T lymphocytes. Monocytes become scavenger receptor-expressing macrophages, uptake oxLDL and become foamy cells before suffering apoptosis. They also release pro-inflammatory cytokines and reactive oxygen species (ROS) to maintain LDL oxidation. With the progression of the condition, smooth muscle and connective tissue proliferate, and dystrophic calcification ensues, leading to the hardening of the atherosclerotic lesion and bulging into the arterial lumen, restricting perfusion. The atherosclerotic lesion is more susceptible to oxidative and mechanical lesions and becomes progressively more fragile, which can culminate in plaque rupture, initiating a hemostatic process that will further decrease perfusion and precipitate the acute atherosclerosis manifestations.

The anti-atherosclerotic effects of propolis are attributed to its ability to modulate both the serum lipoprotein profile and their handling by vascular tissues and infiltrating leucocytes. These properties and respective studies are listed in Table 2.

Propolis administered orally affects the lipoprotein profile in several animal models of atherosclerosis. In lead acetate intoxicated mice, Lybian propolis administrated orally (200 mg/kg) for 30 days was shown to prevent an increase in the serum cholesterol, triglycerides (TG), LDL and very low-density lipoprotein (VLDL) levels, while the HDL levels increased [71]. The same beneficial effect was found for Croatian propolis (50 mg/kg) in high-fat diet-fed mice [70]. These effects seem to be in part attributed to the reversal of cholesterol transport, the expression of ATP-binding cassette transporters A1 and G1 and to the direct stimulation of HDL synthesis [72]. A similar observation was made in healthy Sprague-Dawley rats, where Chinese propolis decreased the hepatic content of cholesterol and TG, besides decreasing the rate of TG hepatic synthesis [66]. Studies carried out in transgenic animals further support these results. In apoprotein E knockout (ApoE^−/−^) mice fed with a high-fat diet, an ethanolic extract of Chinese propolis (160 mg/kg/day for 14 weeks) inhibited atherosclerosis through a combination of cholesterol modulation, inflammation regulation and the inhibition of endothelin and vascular endothelial growth factor (VEGF) secretion [69]. The administration of polyphenols from Brazilian red propolis (250 mg/kg/day) during four weeks to LDL receptor knockout (LDLr^−/−^) mice promoted a decrease in TG, total cholesterol and non-high-density lipoprotein levels [67]. When treated with Brazilian green propolis, rich in artepillin C ((E)-3-(4-hydroxy-3,5-bis[3-methylbut-2-enyl]phenyl)prop-2-enoic acid), pinocembrin ((2S)-5,7-dihydroxy-2-phenyl-2,3-dihydrochromen-4-one) and kaempferol (3,5,7-trihydroxy-2-(4-hydroxyphenyl)chromen-4-one) or with Chilean brown propolis, rich in pinocembrin, caffeic acid phenethyl ester (CAPE, 2-phenylethyl (E)-3-(3,4-dihydroxyphenyl)prop-2-enoate), quercetin (2-(3,4-dihydroxyphenyl)-3,5,7-trihydroxychromen-4-one) and galangin (3,5,7-trihydroxy-2-phenylchromen-4-one), LDLr^−/−^ mice also showed lower non-HDL levels. In mice, artepillin C is also known to act on adipose tissue, promoting the conversion of white adipocytes into brown adipocytes (browning), suggesting a potential role in increasing the energy expenditure and, thus, in preventing/treating metabolic disorders such as obesity and diabetes [73]. A Chinese propolis variety was also found to promote reverse cholesterol transport (i.e., from peripheral tissues back to the liver) by increasing both the serum HDL levels and the expression of efflux transporters ATP-binding cassette transporter A1 and G1, thus restraining the progression of the atherosclerotic lesion [74].

Besides modulating the lipid profile, propolis has also been found to positively interfere with the vascular handling of LDL by plaque macrophages. The advanced and destabilized atherosclerotic plaque exhibits prominent macrophage apoptosis, whereas, when it occurs early in the plaque evolution, it is associated with less cellularity and with slower lesion progression. Ethanolic extracts of Chinese propolis show potential in shielding macrophages from oxidized low-density lipoprotein (oxLDL)-mediated apoptosis. This variety suppressed CD36-mediated oxLDL uptake and the subsequent apoptosis induced by the endoplasmic reticulum stress C/EBP homologous protein (CHOP) pathway [64]. Propolis is also known to interfere with the activity of matrix metalloproteinases. These enzymes are active during tissue remodeling and repair but are also implicated in the atherosclerotic plaque destabilization [65]. Matrix metalloproteinase-9 (MMP-9), in particular, is expressed by plaque macrophages and contributes to its destabilization in both animals and humans [75,76]. The ethanolic extract of Chilean propolis, as well as isolated pinocembrin, was found to display a concentration-dependent inhibitory effect over MMP-9 [65]. Propolis also modulates vascular smooth muscle (VSM) cell proliferation and migration inside the plaque, an effect that was mostly attributed to CAPE [1].

Finally, propolis also slows the progression of atherosclerosis by preventing vascular endothelial dysfunction through its antioxidant, anti-inflammatory and immunomodulatory activities. Chinese and Brazilian propolis show inhibitory activity against phosphatidylcholine-specific phospholipase C in human umbilical vein endothelial cells (HUVECs). This is an important member of the phospholipase C family and thought to be involved in the initiation and progression of atherosclerosis. Furthermore, these varieties also showed important ROS scavenging and nuclear factor kappa B (NF-κB) suppressor effects, which also contribute to their anti-atherosclerotic activity [63].

### 2.2. Anti-Hemostatic Activity

Hemostasis is the set of processes that enable closing off ruptured blood vessels while preventing vascular obstruction (i.e., fibrinolytic activity), as well as maintaining adequate blood rheology [77]. These processes include the spasm of affected blood vessels, platelet plug formation and blood clotting. Once the blood vessels are repaired, fibrinolysis ensues. Upon vessel damage, platelets adhere to the damage site, become activated and attract more platelets in a positive feedback loop via the secretion of several autacoids. Platelets then aggregate to one another via interactions between surface receptors and extracellular ligands to form a hemostatic plug. The clotting process then culminates in the formation of a fibrin matrix, which stabilizes and reinforces the formed plug [78]. The anti-hemostatic activities of propolis and respective studies are listed in Table 3. 

Propolis extracts have demonstrated antiplatelet activity both in vitro and in vivo. Ethanolic extracts from Southeastern Europe samples reduce the adenosine diphosphate (ADP)-induced platelet aggregation when added to blood samples from young patients with thrombophilia, with higher activity being attributed to either low luteolin (2-(3,4-dihydroxyphenyl)-5,7-dihydroxychromen-4-one) content and/or higher pinocembrin-7-methyleter content [81]. Water propolis extracts added to human platelet-rich plasma also inhibited platelet aggregation induced by ADP, the thrombin receptor activator peptide and collagen, with similar results being found for several individual propolis flavonoids, such as galangin [83], pinostrobin ((2S)-5-hydroxy-7-methoxy-2-phenyl-2,3-dihydrochromen-4-one) [83], chrysin (5,7-dihydroxy-2-phenylchromen-4-one) [84] and CAPE, the latter specifically targeting collagen-induced aggregation [83,85]. Propolis also interferes with hemostasis by suppressing the production of plasminogen activator inhibitor-1 (PAI-1), an inhibitor of fibrinolysis that is induced in inflammatory states. The ethanolic extract of Brazilian green propolis suppressed the tumor necrosis factor alpha (TNF-α)-mediated production of PAI-1 in cultured HUVECs [79]. Similar results were found in animal studies. The oral supplementation of mice with an ethanolic extract of Brazilian green propolis for eight weeks attenuated the lipopolysaccharide (LPS)-induced increase in PAI-1 and its plasma activity [80]. Similarly, the intraperitoneal administration of Brazilian green propolis (12.5 mg/kg) also prevented the LPS-mediated increase in PAI-1 [79]. In an in vivo study, the antiplatelet activity has shown to increase the bleeding time in mice receiving Indonesian propolis in a magnitude comparable to that of aspirin (2-acetyloxybenzoic acid) [82]. Propolis also shows an anticoagulant effect indeed potent enough to interfere with the activity of warfarin. CD-1 mice receiving both warfarin (~0.08 mg/day) and an ethanolic extract of Turkish propolis (100 mg/kg/day) for eight days showed lower international normalized ratio (INR) values compared with mice that only received warfarin [86].

### 2.3. Antihypertensive Activity

Hypertension leads to different types of damage to the cardiovascular system, both morphological and functional. Hypertension causes an increase in the arterial shear stress, which damages the endothelium, accelerates atherosclerosis and precipitates the rupture of unstable plaques. Endothelial damage then leads to microhemorrhages, microinfarctions and exudates in the microvascular beds, together with arterial and ventricular remodeling, which combine to cause end-organ damage [87].

The antihypertensive effects of propolis have been studied in different animal models (see Table 4) and have been attributed to several biological activities, including the modulation of vascular tone, anti-inflammatory and antioxidant [88].

In N^ω^-nitro-L-arginine methyl ester (L-NAME)-induced hypertensive Wistar rats, Turkish propolis reduced blood pressure (BP), which was attributed to the ROS scavenging activity of flavonoids, as well as to the presence of arginine, which stimulates nitric oxide synthase (NOS) activity and offsets the NOS-blocking activity of L-NAME. Both of these actions increase the nitric oxide (NO) availability in the VSM, leading to vasorelaxation [94]. Moreover, Uruguayan propolis increased the expression of endothelial nitric oxide synthase (eNOS) and decreased nicotinamide adenine dinucleotide phosphate oxidase (NOX) activity, also increasing the endothelial NO bioavailability [95].

Ethanolic extracts of Turkish propolis displayed a moderate, although nonsignificant, BP-lowering effect in L-NAME-induced hypertensive rats. This effect was attributed to a decrease in tyrosine hydroxylase-mediated catecholamine synthesis [90]. In fact, the co-administration of propolis, pollen and CAPE may mediate a decrease in TH activity in the heart, adrenal glands and hypothalamus of hypertensive rats [96].

In high-salt intake hypertensive rats, Chinese propolis reduced the BP by reducing oxidative stress via a decrease in NOX expression. The consequent increase in endothelial NO bioavailability, together with an anti-inflammatory action (reduction of TNF-α and interleukin (IL)-6 levels) and with an improvement of the myocardial contractile function, ameliorated hypertension in this model [97].

In spontaneously hypertensive rats (SHR), a Brazilian propolis variety, rich in caffeoylquinic acids (CQAs), reduced BP due to its anti-inflammatory action, although a direct vasorelaxant action may also contribute to the response [98]. In another Brazilian propolis variety, the antihypertensive effect was, in fact, attributed to the enhancement of endothelium-dependent vasorelaxation [89]. Whether this effect results from a muscarinic receptor agonistic activity or from the potentiation of its downstream pathway is yet to be clarified. Finally, the combination of a poplar-based Chinese Propolis variety with royal jelly and bee venom reduced blood pressure via a decrease of the serum levels of angiotensin II, endothelin-1 and transforming growth factor-β [99].

In a rat model of renal ablation, the administration of Brazilian red propolis attenuated hypertension, again probably through anti-inflammatory and antioxidant effects [92]. The incorporation of an ethanolic extract of Brazilian propolis in the diet of Otsuka Long-Evans Tokushima Fatty (OLETF) rats during an eight-week period decreased the BP, presumably due to the suppression of metabolic acidosis-induced hypertension [91].

Several randomized clinical trials have investigated the antihypertensive potential of propolis. In a study conducted on healthy subjects, the administration of a commercial propolis supplement (500 mg, twice-daily) resulted in a significant decrease in BP after two months [100]. A double-blind, placebo-controlled study demonstrated that the administration of Beepolis^®^ (Laboratorio Rotterdam Ltda, San Javier, Chile), a 3% Chilean Propolis solution in propylene glycol, decreased BP in hypertensive patients over a period of 90 days [93]. However, in another double-blind, placebo-controlled study, the co-administration of Brazilian Green Propolis (500 mg/day) and antihypertensive drugs to patients with hypertension and chronic kidney disease showed no change in BP after a period of 12 months [88].

Several studies have identified several compounds in propolis with putative vasoactive properties. Several CQAs, namely 3,4-diCQA, 3,5-diCQA and 3,4,5-triCQA, were shown to reduce BP when administered orally at a 10 mg/kg dose, probably due to their anti-inflammatory properties [98]. The flavonoids kaempferide (3,5,7-trihydroxy-2-(4-methoxyphenyl)chromen-4-one), dihydrokaempferide (3,5,7-trihydroxy-2-(4-methoxyphenyl)-2,3-dihydrochromen-4-one), isosakuranetin ((2S)-5,7-dihydroxy-2-(4-methoxyphenyl)-2,3-dihydrochromen-4-one) and betuletol (3,5,7-trihydroxy-6-methoxy-2-(4-methoxyphenyl)chromen-4-one) were found to be the main active substances responsible for the BP-lowering effects of the Brazilian ethanolic propolis in SHRs [101]. Furthermore, CAPE is a major component of European and Asian propolis and displays a vasorelaxant activity in several vascular beds in vitro. This activity is currently attributed to the stimulation of NO release by the endothelium and to hyperpolarizing effects on VSM cells [102]. The flavonoid chrysin has antioxidant effects in L-NAME-induced hypertensive Wistar rats by increasing the activities of glutathione reductase and glutathione-S-transferase [103]. The prevention of cardiac oxidative stress is probably responsible for the complex interplay of the blood pressure-lowering activities, including the decrease in left ventricular function, decrease in plasma angiotensin-II concentration and prevention of NO loss, together with the increase in cardiac hexo oxygenase and cyclic guanosine monophosphate concentrations [104].

### 2.4. Antiangiogenesis Activity

Angiogenesis refers to the tightly controlled and complex process of the formation of new blood vessels from preexisting vessels, essential for tissue development, regeneration and repair [105]. Angiogenesis is initiated when tissues sense a hypoxic environment, responding with the stabilization and translocation of the transcription factor hypoxia-inducible factor 1-alpha (HIF1_α_) to the nucleus, where it induces angiogenic factors such as VEGF. This factor is then released and binds to the VEGF2 receptor on neighbor endothelial cells, inducing cell migration, proliferation and sprouting [106]. Dysfunctional angiogenesis is present in several pathological states, such as inflammation and cancer, leading to persistent and inappropriate blood vessel formation [107,108]. It also contributes to the pathophysiology of atherosclerosis—namely, to the proliferation of the fatty plaque and consequent destabilization, which can lead to intraplaque hemorrhage and plaque rupture. Therefore, antiangiogenic strategies may hinder the progression of the disease [109]. The antiangiogenic activities of propolis and respective studies are listed in Table 5. 

Several propolis varieties—namely, Brazilian, Chilean, Korean and Chinese Red Propolis, have demonstrated antiangiogenic activity in vitro after incubation with HUVECs. Propolis extracts reduce the endothelial cell viability by inducing apoptosis or by suppressing the cellular pathways, leading to cell migration and proliferation [63,92,95,116,118,119,120,121,122]. Rat aortic rings in vitro or animal models of oxygen-induced retinal neovascularization in vivo show that propolis extracts suppress tubulogenesis, an early step in angiogenesis in the embryo, through a reduced accumulation of HIF1_α_ [65,114,116] or by reducing the expression of VEGF [1,110,111,114].

Although, in most propolis varieties, the antiangiogenic activity is attributed to an additive action of dozens of compounds, mainly polyphenols, several isolated propolis compounds are thought to be the main contributors for this activity. Artepillin C, CAPE, galangin, kaempferol and quercetin display important antiangiogenic actions [123], which seemed to be partly related to their antioxidant activity. In fact, flavonols tended to show very strong antiangiogenic activity that seemed to be correlated to their strong antioxidant activity, while flavones possessed relatively high antiangiogenic activity, with only a limited degree of antioxidant activity [123]. In addition, CQA derivatives are thought to possess substantial antiangiogenic activity [111].

### 2.5. Endothelial-Protecting Activity

The endothelium is the innermost vascular layer and pivotal in the orchestration of the regulation of cardiovascular function, being involved in the modulation of vascular tone, maintaining blood rheology, regulating inflammatory and immune response and neovascularization [124]. As a physical barrier, the endothelium is also the first vascular defense system against a vast number of aggressions, including mechanical (i.e., shear stress), chemical and biological [125]. The endothelium is considered functional when it is highly capable of secreting NO, which displays important anti-atherosclerotic and anti-hemostatic activities, besides its vasorelaxant action. Oxidative stress—for example, arising from tissue inflammation—leads to the accumulation of ROS and reactive nitrogen species (RNS). Among the latter, peroxynitrite leads to a decrease in NO availability by both direct and indirect actions. Firstly, peroxynitrite directly decreases NO availability by directly reacting with it. Secondly, it oxidizes the tetrahydrobiopterin (BH_4_) cofactor and leads to eNOS uncoupling, which further increases superoxide production. Finally, peroxynitrite also promotes protein nitration, contributing further to the dysfunction and death of endothelial cells [126,127]. Other pathophysiological stimuli, such as hyperglycemia, hyperlipidemia, oxLDL, hypertension [128], mental stress [129], aging [130] and exposure to specific drugs [131], can disrupt the molecular mechanisms regulating NO bioavailability. The decrease in the endothelial capacity for NO release is termed endothelial dysfunction and constitutes a main hallmark of several cardiovascular diseases [132].

Several studies detail the antioxidant effect of propolis on endothelial cells in vitro, with the vast majority using Chinese propolis varieties. The pretreatment of HUVECs with an ethanolic extract of Chinese propolis improved the oxLDL-mediated oxidative stress by decreasing NOX activation, as well as ROS and malonaldehyde generation, while increasing antioxidant enzyme activities [69]. Additionally, this variety reduced the oxLDL uptake by HUVECs by attenuating the oxLDL-induced upregulation of lectin-like oxidized low-density lipoprotein receptor-1 (LOX-1), a critical receptor in this process [133]. Furthermore, cell viability was maintained by increasing the release of lactate dehydrogenase, by the activation of caspase-3 and by oxLDL-induced cell apoptosis in a dose-dependent way, suggesting a protective role against oxLDL-mediated endothelial dysfunction [64]. The Chinese poplar propolis protected against oxLDL-mediated endothelial dysfunction by several mechanisms, including activating the PI3K/Akt/mTOR signaling pathway, by inhibiting the LOX-1/p38 mitogen-activated protein kinase (MAPK) level, by depressing ROS production and protecting MMPs to inhibit apoptosis and autophagy [134]. Chinese propolis protects the endothelium against lipopolysaccharide-mediated endothelial dysfunction by suppressing autophagy and blocking the MAPK/NF-κB signaling pathway [135]. Hyperglycemia is another factor responsible for endothelial dysfunctional, causing the many microvascular and macrovascular complications that accompany diabetes [136]. Chinese propolis prevented the glucose-induced impairment of phenylephrine-induced contraction and endothelial-dependent vasorelaxation. Moreover, it increased the superoxide dismutase (SOD) activity and glutathione (GSH) concentration while decreasing the concentration of malonaldehyde, suggesting an important protective role against glucose-induced vascular endothelial dysfunction [137].

### 2.6. Myocardial-Protecting Activity

Cardiomyocytes, endocardial and coronary endothelial cells, as well as cardiac nerves, are all sources of NO. This mediator is necessary for normal cardiac physiology and has a protective role in heart ischemia. Oxidative and nitrosative stress are also known to promote cardiomyocyte dysfunction. Peroxynitrite impairs cardiac function through multiple mechanisms, including the activation of nuclear enzyme poly(ADP-ribose) polymerase (PARP) and of MMPs, a family of enzymes involved in protein degradation in the extracellular matrix, especially collagen and elastin. Nitrosative stress also promotes the S-nitrosylation of cysteine residues within multiple proteins and can perturb their functions [138]. Nitrosative stress is involved in the pathogenesis of acute heart failure, congestive heart failure [139,140] and coronary heart disease [118].

The pretreatment of cardiomyocytes with Malaysian propolis improved the adverse histological effects of isoproterenol-induced myocardial infarction in mice mainly through its ROS scavenging activity. A pretreatment with propolis suppressed the cardiac markers and enhanced the histopathological result through its radical-scavenging activity and potentially lipid peroxidation inhibition [55]. Several varieties of Chinese propolis have also displayed antioxidant activity against hydrogen peroxide-induced oxidative injury in rat cardiomyocytes. From all the analyzed compounds, CAPE, benzyl caffeate (BZC, benzyl (E)-3-(3,4-dihydroxyphenyl)prop-2-enoate) and cinnamylcaffeate (CNC, ([E]-3-phenylprop-2-enyl) [E]-3-(3,4-dihydroxyphenyl)prop-2-enoate) showed higher cytoprotective effects, followed by chrysin, pinobanksin ((2R,3R)-3,5,7-trihydroxy-2-phenyl-2,3-dihydrochromen-4-one) and 3,4-dimethoxycinnamic acid (DMCA, (E)-3-(3,4-dimethoxyphenyl)prop-2-enoic acid). Caffeic acid phenethyl ester, BZC and CNC improved the cellular antioxidant capacity by reducing the malonaldehyde levels and increasing the SOD and glutathione peroxidase activities, reducing the intracellular calcium ion levels and preventing cell apoptosis [119]. Few studies concerning the effect of propolis on heart electrical activity are available, being rather centered on CAPE and pinocembrin. These compounds show cardioprotective effects in ischemia-reperfusion animal models—namely, decreasing the extension of the infarction, as well as preventing arrhythmias [121,141,142].

### 2.7. Antioxidant and Anti-Inflammatory Activities

The accessible information shows that, no matter its geographical origin and composition, propolis displays important antioxidant activities. In certain varieties, however, there seems to be a close relation between the antioxidant activity and the concentration of particular substances. For example, the antioxidant activity of Chinese red propolis is primarily attributed to CAPE [110], in Chilean propolis, it is mainly attributed to its phenylpropanoid content [143,144] and, in Brazilian red propolis, it is ascribed to chalcones and isoflavonoids, including 7-O-methylvestitol (5-methoxy-2-([3S]-7-methoxy-3,4-dihydro-2H-chromen-3-yl)phenol), medicarpin ((6aR,11aR)-9-methoxy-6a,11a-dihydro-6H-(1)benzofuro(3,2-c)chromen-3-ol) and 3,4,2′,3′-tetrahydrochalcone ((E)-1-(2,3-dihydroxyphenyl)-3-(3,4-dihydroxyphenyl)prop-2-en-1-one) [17]. Moreover, in Brazilian red propolis, the total flavonoid content is associated with antioxidant activity, implying that all the phenolic and flavonoid substances present contribute to this activity [145]. In relation to this antioxidant impact, bioactive compounds in propolis affect many biochemical signaling pathways and, thus, physiological and pathological procedures. An antioxidant capacity is one of propolis’ most significant properties [1]. Even though several experiments confirm the prospective antioxidant activity of propolis, no solid information is available on the safe dose in humans. Clinical studies using propolis and its biologically active compounds, including safety and bioavailability research, are therefore needed.

Besides the antioxidant activity observed in vitro for different propolis varieties, several in vivo studies have been performed in humans to assess the impact of propolis supplementation on the general antioxidation status. A commercially available propolis sample was administered for 30 days (48.75 mg flavonoids/day) to a group of healthy subjects. In males, a transient decrease in malonaldehyde level was observed, together with an increase in SOD activity, while no effect was observed in females. These results suggest the possibility that propolis only has a transient and sex-dependent effect on serum lipid peroxidation [146].

Randomized clinical trials have been conducted in type 2 diabetes mellitus patients. A supplementation with Brazilian green propolis (900 mg/day, 18 weeks) was associated with the increase of serum levels of GSH and total polyphenols and with the decrease of serum carbonyls (i.e., protein oxidation markers), as well as of lactate dehydrogenase activity [147]. The application of a propolis spray to diabetic foot ulcers (Beepolis^®^ in 3% in propylene glycol, eight weeks or until cicatrization) was also associated with the increase of serum GSH levels, together with the decrease of TNF-α and IL-10 levels [148].

## 3. Conclusions

Propolis is a folk medicine with long-standing popularity. To the authors’ knowledge, this is the first paper to provide a comprehensive review centered only on the cardiovascular effects of propolis, with the most recent literature having been considered. The reviewed studies affirm the interest of using propolis as a natural agent capable of counteracting the oxidative stress and inflammation that underlie several cardiovascular diseases, such as hypertension and atherosclerosis. Several varieties with cardiovascular effects were identified (see Table 6), with Brazilian green and red varieties showing the largest number of beneficial activities. Several factors are known to affect propolis composition, thus hindering the selection of a given variety for a specific purpose. Further research, especially preclinical, should be conducted to assess the cardiovascular benefits of given varieties with different compositions. Although most of the cardiovascular beneficial effects seem to result from the combined activities of many compounds acting through different signaling pathways, promising results have also been noted with several individual compounds that could integrate the current therapeutic arsenal. The possibility of using propolis as a food supplement should also be considered.

## Figures and Tables

**Table 1:** Origins and major constituents of propolis (adapted from [6,7,8]).

Authors	Geographical Origin	Major Constituents
Piccinelli et al. (2013) [9]; Soltani et al. (2017) [10]	Algeria	Diterpenes
Salas et al. (2016) [11]; Fangio et al. (2019) [12]	Argentina	Sesquiterpenes, flavonoids, phenolic acids
Huang et al. (2014) [13]; Silva-Carvalho et al. (2015) [4]	Australia	Stilbenes, phenylpropanoids, flavonoid esters
Trusheva (2006) [14]; Alencar et al. (2007) [15]; Silva et al. (2008) [16]; Righi et al. (2011) [17]	Brazil (red)	Triterpenoids, isoflavonoids, prenylated benzophenones
Paviani et al. (2013) [18]; Quintino et al. (2020) [19]	Brazil (green)	Triterpenoids, flavonoids, phenolic acids
da Silveira et al. (2016) [20]; Machado et al. (2016) [21]	Brazil (yellow)	Triterpenoids
Fernandes et al. (2015) [22]; Lima et al. (2019) [23]	Brazil (brown)	Sesquiterpene, flavonoids, phenolic acids
Papachroni et al. (2015) [24]	Cameroon	Triterpenoids
	Congo	
Montenegro et al. (2001) [25]; Muñoz et al. (2001) [26];	Chile	Benzophenones, flavonoids, diterpenes
Silva-Carvalho et al. (2015) [4]; Miguel and Antunes (2011) [27]	China	Flavonoids
Graikou et al. (2016) [28]	Croatia	Diterpenes
Bankova (2005) [29]	Cuba	Polyisoprenylated benzophenones
Graikou et al. (2016) [28]	Cyprus	Diterpenes
Hegazi et al. (2002) [30]	Egypt	Di- and triterpenes
Rushdi et al. (2014) [31]	Ethiopia	Triterpenoids
Velikova et al. (2000) [32]; Bankova et al. (2002) [33]; Melliou and Chinou (2004) [34];	Italy	Diterpenic and phenolic compounds
Huang et al. (2014) [13]; Silva-Carvalho et al. (2015) [4]	Iran	Monoterpenes, diterpenes, sesquiterpenes, coumarins
Banksota et al. (2001) [35]; Huang et al. (2014) [13]	Japan	Prenylated flavanones
Petrova et al. (2010) [36]; Popova et al. (2011) [37]	Kenya	Triterpenoids
Huang et al. (2014) [13]; Toreti et al. (2013) [38]; Silva-Carvalho et al. (2015) [4]	Korea	Lignans
Siheri et al. (2016) [39]; Siheri et al. (2014) [40]	Lybia	Diterpenes
Popova et al. (2011) [37]	Malta	
Martinotti (2015) [41]	Mexico	Prenylated benzophenones
Popova et al. (2015) [42]; El-Guendouz et al. (2016) [8]; Miguel (2013) [43]	Morocco	Diterpenes, flavonoids, phenolic acid esters
Huang et al. (2014) [13]	Nepal	Sesquiterpenoids, flavonoids and neo-flavonoids
Huang et al. (2014) [13]; Silva-Carvalho et al. (2015) [4]	Poland	Flavonoids, phenolic acids
Huang et al. (2014) [13]; Silva-Carvalho et al., (2015) [4]; Miguel (2013) [43]	Portugal	
Inui et al. (2012) [44]	Solomon	Prenylated flavonoids, stilbenes
Bankova et al. (2002) [33]	Switzerland	Terpenoids
Bankova (2005) [29]; Martinotti (2015) [41]	Taiwan	C-prenyl-flavanones
Miguel (2013) [43]	Tunisia	Flavonoids, phenolic acids
Popova et al. (2005) [45]; Doganli (2016) [46]	Turkey	Diterpenes, flavonoids, phenolic acids
Bankova (2005) [29]	Venezuela	Polyisoprenylated benzophenones

**Table 2:** Description and main results of the most relevant studies assessing the anti-atherosclerotic activity of propolis.

Authors	Variety/Substance	Dose/Concentration and Duration of Treatment	Study Type	Experimental Model	Main Results
Xuan et al. (2014) [63]	Ethanolic extracts of Chinese or Brazilian green propolis	12.5 μg/mL	In vitro	Human umbilical vein endothelial cell culture	Inhibition of phosphatidylcholine-specific phospholipase C reactive oxygen species scavengingSuppression of nuclear factor kappa B pathway
Tian et al. (2015) [64]	Ethanolic extract of Chinese propolis	7.5, 15 and 30 mg/L	In vitro	RAW264.7 macrophage culture	Suppression of CD36-mediated oxidized low-density lipoprotein uptake subsequent apoptosis of plaque macrophages
Saavedra et al. (2016) [65]	Ethanolic extract of Chilean propolis	1, 2.5, 5.0, 7.5, 10 μg/mL	In vitro	RAW264.7 macrophage culture	Concentration-dependent inhibition of matrix metalloproteinase-9
Li et al. (2012) [66]	Ethanolic extract of Chinese propolis	50, 100 or 200 mg/kg/day for 10 weeks	In vitro	High-fat diet-induced type 2 diabetic mice	Decrease in the hepatic content of cholesterol and triglycerides. Decrease in rate of hepatic synthesis of triglycerides
Daleprane et al. (2012) [67]	Ethanolic extract of Brazilian green, red and brown propolis	250 mg/kg per day for 4 weeks	In vivo	Low-density lipoprotein receptor knockout mice receiving a cholesterol-enriched diet to induce atherosclerotic lesions	Reduction of atherosclerotic lesions through modulation of inflammatory and angiogenic factors (highest activity for red propolis)
Azab et al. (2013) [68]	Aqueous extract of Lybian propolis	200 mg/kg for 30 days	In vitro	Lead acetate intoxicated male albino mice	Prevention of the increase in serum cholesterol, triglycerides, low-density lipoprotein and very low-density lipoprotein, together with the increase in high-density lipoprotein
Fang et al. (2013) [69]	Ethanolic extract of Chinese propolis	160 mg/kg/day for 14 weeks	In vitro	Apoprotein E knockout mice fed a high-fat diet	Inhibition of atherosclerosis through cholesterol modulation, inflammation regulation and inhibition of endothelin and vascular endothelial growth factor secretion
Oršolić et al. (2019) [70]	Ethanolic extract of Croatian propolis	50 mg/kg for 30 days	In vitro	High-fat diet-fed mice	Prevention of the increase in serum cholesterol, triglycerides, low-density lipoprotein and very low-density lipoprotein, together with the increase in high-density lipoprotein

**Table 3:** Description and main results of the most relevant studies assessing the anti-hemostatic activity of propolis.

Authors	Variety/Substance	Dose/Concentration and Duration of Treatment	Study Type	Experimental Model	Main Results
Ohkura et al. (2012) [79]	Ethanolic extract of Brazilian green propolis	0.5% (*w*/*w*)	In vitro	Human umbilical vein endothelial cell (HUVEC) culture incubated with tumor necrosis factor alpha	Suppression of tumor necrosis factor alpha-mediated secretion of plasminogen activator inhibitor-1
Ohkura et al. (2016) [80]	Ethanolic extract of Brazilian green propolis	0.5% (*w*/*w*) in medium fat diet for 2, 4 or 8 weeks	In vitro	Lipopolysaccharide-induced inflammation in Kwl:ICR mice	Attenuation of lipopolysaccharide-mediated secretion of plasminogen activator inhibitor-1 and of its plasma activity after 8 weeks treatment
Bojic et al. (2018) [81]	Ethanolic extracts from Croatian, Macedonian and Bosnian propolis	Different concentrations until reaching the minimal antiaggregatory concentration	In vitro	Whole human blood	Reduction in adenosine diphosphate-induced aggregant effect, with minimal antiaggregatory concentrations ranging from 5 μM to 10.4 mM
Ohkura et al. (2012) [79]	Ethanolic extract of Brazilian green propolis	12.5 mg/kg administered intraperitoneally for 7 days	In vivo	Lipopolysaccharide-induced inflammation in Kwl:ICR mice	Attenuation of lipopolysaccharide-mediated secretion of plasminogen activator inhibitor-1 and of its plasma activity
Martina et al. (2018) [82]	HDI Origins™ Bee Propolis (Indonesian propolis)	65 mg/kg/day administered orally for 12 days	In vivo	Healthy Double Distsch Webster mice	Increase in bleeding time

**Table 4:** Description and main results of the most relevant studies assessing the antihypertensive activity of propolis.

Authors	Variety/Substance	Dose/Concentration and Duration of Treatment	Study Type	Experimental Model	Results
Kubota et al. (2004) [89]	Ethanolic extract of Brazilian propolis	0.5% (*w*/*w*) for 4 weeks	In vitro	Spontaneously hypertensive rats	Potentiation of acetylcholine-dependent aortic vasorelaxation
Gogebakan et al. (2012) [90]	Ethanolic extract of Turkish propolis	200 mg/kg orally administered in the last 5 days of the study	In vivo	Wistar rats with hypertension following N^ω^-nitro-L-arginine methyl ester -administration (15 days)	Tyrosine hydroxylase-mediated decrease in catecholamine synthesis
Aoi et al. (2013) [91]	Ethanolic extract of Brazilian propolis	0.1% and 0.5% (*w*/*w*) for 8 weeks	In vivo	Otsuka Long-Evans Tokushima Fatty rats	Blood pressure decrease
Teles et al. (2015) [92]	Ethanolic extract of Brazilian red propolis	150 mg/kg/day for 60 days	In vivo	5/6 renal ablation model in Wistar rats	Attenuation of blood pressure increase
Mujica et al. (2017) [93]	Propyleneglycolic extract of Chilean propolis	3% (*w*/*w*) orally administrated (15 drops, twice daily) for 90 days	In vivo	Hypertensive patients	Blood pressure decrease

**Table 5:** Description and main results of the most relevant studies assessing the anti-angiogenic activity of propolis.

Authors	Variety/Substance	Dose	Study Type	Experimental Model	Main Results
Izuta et al. (2009) [110]	Ethanolic extract of Chinese red propolis	0.3–3.0 μg/mL	In vitro	HUVEC culture	Suppression of vascular endothelial growth factor-induced HUVEC proliferation and migration
Chikaraishi et al. (2010) [111]	Aqueous extract of Brazilian green propolis	100 mg/mL	In vitro		Suppression of vascular endothelial growth factor-induced HUVEC proliferation, migration, and tube formation, attributed to caffeoylquinic acids
Kunimasa et al. (2011) [112]	Ethanolic extract of Brazilian green propolis	6.25, 12.5 and 25 μg/mL	In vitro		Concentration-dependent induction of apoptosis in tube-forming HUVECs
Meneghelli et al. (2013) [113]	Hydro-alcoholic extract of Brazilian propolis	100, 130, 150 and 180 μg/mL	In vitro		Significant decrease in cell viability, proliferation, migration and in capillary tube formation
		450 mg/kg	In vivo	Chick embryo chorioallantoic membrane assay	Inhibition of angiogenesis and vasculogenesis
Cuevas et al. (2014) [114]	Ethanolic extract of Chilean propolis	250 mg/kg	In vitro	Aortic rings from low-density lipoprotein receptor knockout male mice	Decreased expression of vascular endothelial growth factor A
Cuevas et al. (2015) [115]	Ethanolic extract of Chilean propolis	1–15 μg/mL	In vitro	HUVEC culture	Attenuation of migration and sprouting
		15 μg/mL	In vitro	Aortic rings from male Wistar rats	Inhibition of hypoxia-inducible factor 1 alpha accumulation in a concentration-dependent manner
Daleprane et al. (2012) [116]	Polyphenolic extract from red propolis	10 mg/L	In vitro	EA.hy926 cell culture	Angiogenesis attenuation; inhibition of hypoxia-induced expression of vascular endothelial growth factor; decrease in hypoxia-inducible factor 1 alpha accumulation
			In vitro	Aortic rings from male Wistar rats	Sprouting of endothelial cell tubular structures
Park et al. (2014) [117]	Ethanolic extract of Korean propolis	6.25, 12.5, or 25 μg/mL	In vitro	HUVEC culture	Inhibition of proliferation of HUVECs and tube formation
Chikaraishi et al. (2010) [111]	Aqueous extract of Brazilian green propolis	300 mg/kg/day administered subcutaneously for 5 days	In vivo	Oxygen-induced retinopathy in C57BL/6 mice	Suppression of retinal neovascularization, attributed to caffeoylquinic acids
Daleprane et al. (2012) [116]	Polyphenolic extract from red propolis	10 mg/L	In vivo	Chick embryo chorioallantoic membrane assay	Reduced angiogenesis
Park et al. (2014) [117]	Ethanolic extract of Korean propolis	6.25, 12.5 or 25 μg/egg	In vivo	Chick embryo chorioallantoic membrane assay	Reduced angiogenesis

**Table 6:** Propolis activities according to their geographical origin (N.F.—not found and X—scientifically proven activity).

Origin	Endothelial and Myocardial Protective	Anti-Hypertensive	Anti-Atherosclerotic	Anti-Hemostatic	Anti-Angiogenic
Argentina	N.F.	N.F.	X [149]	N.F.	N.F.
Brazil (green propolis)	X [147]	N.F.	N.F.	N.F.	X [111,112]
Brazil (red propolis)	X [17,145]	X [88,92]	X [67]	N.F.	N.F.
Chile	X [143]	X [93]	X [65]	N.F.	X [115]
China	X [69,119]	X [99]	X [67,70,71]	N.F.	N.F.
China (red propolis)	X [110]	N.F.	X [72]	NF	X [110]
Indonesia	N.F.	N.F.	N.F.	X [82]	N.F.
Korea	N.F.	N.F.	N.F.	N.F.	X [117]
Malaysia	X [55]	N.F.	N.F.	X [150]	N.F.
Turkey	N.F.	X [88,90]	N.F.	N.F.	N.F.
